# On the Feasibility of SERS-Based Monitoring of Drug Loading Efficiency in Exosomes for Targeted Delivery

**DOI:** 10.3390/bios15030141

**Published:** 2025-02-23

**Authors:** Jun Liu, Siddharth Srivastava, Tieyi Li, Faycal Moujane, John Y. Lee, Yiqing Chen, Huinan Liu, Sophie X. Deng, Ya-Hong Xie

**Affiliations:** 1 Department of Materials Science and Engineering, University of California Los Angeles, Los Angeles, CA 90095, USA; 2 Cornea Division, Stein Eye Institute, University of California Los Angeles, Los Angeles, CA 90095, USA; 3 Department of Bioengineering, University of California Riverside, Riverside, CA 92521, USA

**Keywords:** surface-enhanced Raman scattering (SERS), single vesicle analysis, exosomes, drug loading quantification, osmotic pressure control

## Abstract

Cancer, a significant cause of mortality, necessitates improved drug delivery strategies. Exosomes, as natural drug carriers, offer a more efficient, targeted, and less toxic drug delivery system compared to direct dispersal methods via ingestion or injection. To be successfully implemented as drug carriers, efficient loading of drugs into exosomes is crucial, and a deeper understanding of the loading mechanism remains to be solved. This study introduces surface-enhanced Raman scattering (SERS) to monitor drug loading efficacy at the single vesicle level. By enhancing the Raman signal, SERS overcomes limitations in Raman spectroscopy. A gold nanopyramids array-based SERS substrate assesses exosome heterogeneity in drug-loading capabilities with the help of single-layer graphene for precise quantification. This research advances targeted drug delivery by presenting a more efficient method of evaluating drug-loading efficiency into individual exosomes through SERS-based monitoring. Furthermore, the study explores leveraging osmotic pressure variations, enhancing the efficiency of drug loading into exosomes.

## 1. Introduction

### 1.1. Drug Delivery

Cancer remains a leading cause of mortality worldwide, with over 1.9 million new cases diagnosed and 609,360 deaths reported in the United States in 2022 alone [1]. Moreover, cancer imposes a significant financial burden, with NIH data estimating cancer care costs at $183 billion in 2015, projected to rise 34% to $246 billion by 2030, primarily due to population growth [2]. Scientists and doctors have been fighting cancers since Marie Curie and Pierre Curie discovered radiation at the end of the 19th century for over 125 years [3]. The two pursued efficient strategies to combat cancers which are early cancer detection and more effective therapeutics, have emerged as consensus in the field [4,5,6,7]. Traditional chemotherapy faces challenges such as systemic toxicity and low targeting efficiency, necessitating the development of precise drug delivery systems. Exosome-mediated drug delivery has gained significant attention due to its biocompatibility, ability to traverse biological barriers, and potential for tumor-targeted therapy [8,9,10]. Achieving target specificity in cancer therapy is essential. For instance, the toxicity of chemotherapy to healthy cells causes significant patient morbidity. Exosome-encapsulated drugs offer a promising solution by enabling targeted delivery through customized surface receptors and protecting drug integrity, potentially reducing nonspecific activity and improving bioavailability.

### 1.2. Exosomes

Exosomes, small extracellular vesicles secreted by most cell types (typically ranging from 30 to 200 nm in diameter), have emerged as highly promising carriers for drug delivery and imaging platforms [11]. Due to their natural abilities to carry various biological molecules [12,13] from parental cells and to traverse biological barriers like the blood-brain barrier [14], exosomes facilitate intercellular cell-to-cell communication [11,15] and deliver therapeutic cargoes with high specificity and minimal immunogenicity [16]. Furthermore, the inherent biocompatibility of exosomes makes them ideal candidates for targeted drug delivery in therapeutic applications, including cancer treatment [17,18]. Their potential as natural drug carriers is being extensively explored for targeted therapy [19], offering advantages such as deep tissue penetration, prolonged circulation due to negative zeta potential, and membrane compatibility [12,20]. Zhang et al. demonstrated that neutrophil-derived exosomes triggered tumor cell apoptosis by delivering proteins with cytotoxic effect and they further enhanced the targeting ability of exosomes by enhancing the surface with superparamagnetic materials [21]. Kim et al. exploited cancer-derived exosomes from ovarian cancer cell line SKOV3 and HEK293 as delivery vehicles for CRISPR/Cas9, to specifically target cancer cells, leveraging the cancer cell-specific tropism of these exosomes, which led to the triggering of programmed cell death in ovarian cancer cells [22]. Zhao et al. examined the use of exosomes derived from autologous breast cancer cells, with a natural affinity for lung tissue, as delivery vehicles for siRNA (siS100A4), which is capable of suppressing the expression of the S100A4 gene and preventing the initiation of tumor growth, to successfully target the pre-metastatic niche in the lungs [18]. Exosomes hold great superiority as pure natural carriers over other nanocarriers such as liposomes and polymetric nanoparticles. Aside from being ubiquitously distributed in the human body fluid circulation system, exosomes possess outstanding biocompatibility and higher stability in blood over their carrier competitors. By loading various therapeutic molecules or drugs into exosomes, there exists a potential for highly efficient medical treatment with fewer side effects on healthy cells. Many researchers have successfully demonstrated the feasibility of loading substances into exosomes, including nucleic acids, proteins, and small molecules.

### 1.3. Current Technology of Drug Loading into Vesicles

Numerous loading techniques have been developed for drug loading into exosomes over the past few decades. Based on the mechanism, they can be categorized into passive and active loading [23]. Passive loading involves incubation [24], transfection of donor cells [17], and freeze-thaw cycles [25]. On the other hand, active loading techniques incorporate more options for researchers, including sonication [26], extrusion [27], electroporation [28], saponin-assisted permeabilization [27], click chemistry [29], ligand-displaying method [30], and other novel engineered EVs-based platforms [12]. For more specific and precise information as well as the comparison among various loading methods, please refer to these review articles [12,23,31].

In this work, we enhanced the loading efficiency of doxorubicin (DOX) into exosomes by combining incubation with osmotic pressure control. DOX, a widely used chemotherapeutic agent, was chosen as the loading target into exosomes in our study [28,32]. The DOX used in the study came in as a hydrophilic doxorubicin hydrochloride (DOX·HCl) compound which tends to be encapsulated into the internal aqueous compartments of exosomes due to their aqueous interior [23]. Incubation allows drugs to diffuse directly into exosomes without specialized equipment and is widely used for loading various agents, including chemotherapeutics. However, it often results in low efficiency and uneven drug distribution [23]. Osmotic pressure control involves exposing exosomes to a hypotonic solution, which has a lower concentration of non-penetrating solutes compared to the intracellular fluid, to improve loading efficiency [33,34]. This combined approach was validated using our SERS platform. This osmotic imbalance causes water to flow into the cells or vesicles through osmosis leading to swelling of the entities (cells or vesicles) and increased membrane permeability. The process enables the entities to be more permeable to certain substances, which can facilitate the entry of molecules that would otherwise not easily penetrate the cell or vesicle membrane. This strategy requires careful experimental optimization to balance enhanced drug loading with the preservation of exosome structure and function, and thus, has not been studied as intensively as other active loading techniques, such as sonication, and electroporation. Nonetheless, researchers have applied this technique to enhance drug loading into exosomes with success [35,36]. Nair et al. presented an approach that combines cytochalasin B treatment with hypo-osmotic stimulation to increase both the production and drug loading capacity of EVs [35]. Lee et al. successfully demonstrated that tonicity control of drug loading into exosomes outperformed conventional techniques like sonication and extrusion by roughly 4 and 7 times, respectively [36].

### 1.4. Scope of Study

In this work, we proposed to build a monitoring technology for enhanced drug loading of exosomes by Surface-enhanced Raman scattering (SERS) identification of molecules. Our technique provides statistical and biochemical information on a single vesicle level, which can help in the development of diagnostic and therapeutic tools for many diseases.

Raman spectroscopy or Raman scattering, as the foundation of our technology, is regarded as a powerful and versatile analytical tool applied in multiple fields for the detection, identification, and characterization of molecules. It works by collecting vibrational modes of the chemical bonds present in the specimen, which pattern from the inelastic scattering of photons by matter. The scattering process can be described simplified as photons from a monochromatic laser source being incident on molecules and interacting with the electric dipole of the molecules which results in a shift in the frequency of the scattered light. The scattered lights with various shifts are then collected by a detector to generate the spectrum over a certain Raman shift range. There are many advantages of Raman spectroscopy, such as being label-free, non-destructive, capable of molecule fingerprinting, and extreme versatility. However, it suffers from several serious hindrances that restrict its wide utilization. The low signal-to-noise ratio is one of the greatest challenges in Raman spectroscopy, which makes it incompetent to detect weak Raman signals for high sensitivity requirements. The poor signal-to-noise ratio of Raman spectroscopy roots in the low probability of inelastic photon scattering occurrence, which is estimated to be only a micro-fraction of the photons, about 1 in 10^6^~10^8^, that can be scattered inelastically.

SERS, on the other hand, as the improved methodology derived from Raman scattering, has solved this adverse flaw by amplifying the signal-to-noise ratio up to a factor of 10^14^. SERS enables the interaction among the incident light, the nanometal surface, and the adsorbed molecules on the surface that amplify the Raman signal to a significant level. It has been studied extensively for biosensing of both small and large specimens, such as viruses, amino acids, peptides, nucleic acids, extracellular vesicles, and cells. Owing to its high sensitivity and fingerprinting capabilities, researchers have successfully brought this technique to the level of single-molecule detection and characterization. The precisely designed and fabricated nano metal surface of a SERS substrate, which provides the so-called “hotspots” of electromagnetic (EM) field boosting, is the key to generating a high signal-to-noise ratio and uniform detectability of the specimen. Our group designed and fabricated a gold nanopyramids array-based substrate for SERS applications, where hotspots are on the side surface of the pyramid along the direction of the electromagnetic field. Promising results have been achieved in previous work, for instance, detection and characterization of exosomes [37], amyloid-β (Aβ42) peptide [38], and COVID virus [39].

In this work, we established a SERS-based monitoring technology specifically for the characterization of drug loading into exosomes. Currently, the mainstream characterization methods for drug loading into exosomes can be categorized into two groups: the evaluation of loading efficiency and the effect of drug loading on exosomes. The former mainly involves the usage of ultraviolet-visible (UV-Vis) spectroscopy, high-performance liquid chromatography (HPLC), and reverse transcription-polymerase chain reaction (RT-PCR) in the determination of drug loading efficiency into exosomes. The latter emphasizes the effect on exosomes after loading of drugs, such as size distribution changes, morphological and structural changes, and chemical compositional changes. Multiple techniques can be adopted for this characterization, including nanoparticle tracking analysis (NTA), transmission electron microscopy (TEM), and flow cytometry. Specifically, the techniques for evaluation of loading efficiency are either direct measurements of loading efficiency or are based on a bulk sample of exosomes in the process. Exosomes are well known to maintain high heterogeneity in size, cargo, and function during the secretion process. Allowing the study or quantification of drug loading on a single vesicle level provides a chance to gain a deeper understanding of their heterogeneity and the impact on their biological functions. Our group previously demonstrated the single-molecule sensitivity of our SERS substrate [40], underscoring its capability of detecting individual exosomes. The gold nanopyramid SERS substrate features hotspots approximately 100 nm in size, aligning with the dimensions of small exosomes and facilitating single-exosome detection [37,41,42]. This alignment ensures that the SERS signals predominantly originate from individual exosomes rather than aggregates. Theoretical calculations, detailed in the Supplementary Materials, indicate that with an initial concentration of 5 × 10^8^ particles/mL and a 5 μL droplet drying into a 5 mm diameter spot, the average spacing between exosomes is about 2.8 μm. However, our SERS measurements typically reveal an average spacing exceeding 5 μm, which is significantly larger than both the exosome size and the 1 μm laser spot size employed during measurements. This discrepancy is likely due to some exosomes being obscured by salts precipitated during the drying process. The substantial spacing supports the notion that each spectrum corresponds to a single vesicle. Our spectral acquisition protocol involves an initial coarse SERS scan over a large area with a 10 μm step size, followed by a finer local scan with a 1 μm step size to obtain high-quality spectra. This methodology enhances the precision of single-vesicle detection by ensuring that the collected spectra pertain to individual vesicles rather than aggregates. Collectively, these strategies provide robust evidence for single-vesicle detection, enabling systematic isolation and analysis of individual vesicles, thereby enhancing the accuracy and reliability of our SERS measurements. Further, analyzing individual exosome SERS spectra can uncover variations in their drug-loading capacities, revealing that certain subpopulations may contribute more significantly to this process. This insight could enhance the development of exosomes as therapeutic tools for disease treatment. To enable precise quantification of drug loading, a single-layer graphene mask was applied to the SERS substrate, serving as an internal gauge for the local electromagnetic (EM) field. This graphene layer is crucial for accurate quantification due to two primary factors. First, the surface plasmon resonance on the gold substrate is inherently non-uniform [40,43]. For example, the EM field generated at the shoulder position of a gold nanopyramid is significantly stronger than that at a valley or peak position. This variability necessitates a method to account for these differences. Second, the intensity of SERS peaks is a convoluted result of both local molecular density and local EM field intensity. By introducing single-layer graphene, its G peak in the spectra provides a direct measurement of the local EM field intensity. This enables deconvolution of the SERS signal for accurate quantification of the drug molecules. Additionally, simulations of the electromagnetic field using the finite-difference time-domain (FDTD) method were performed to further validate and illustrate the role of the local EM field in quantification. These simulations, which detail the field distribution across the substrate, are included in the Supplementary Materials to provide additional context and support for this approach.

### 1.5. Mathematical Model of Osmosis-Assisted Loading

When exosomes are exposed to hypotonic conditions, they undergo swelling due to osmotic pressure, which can eventually lead to membrane rupture. Before rupturing, these vesicles may experience stages where their membranes become leaky, yet they still maintain biological viability. Our objective is to identify and exploit this specific window to achieve optimal loading efficiency.

**Figure 1:**
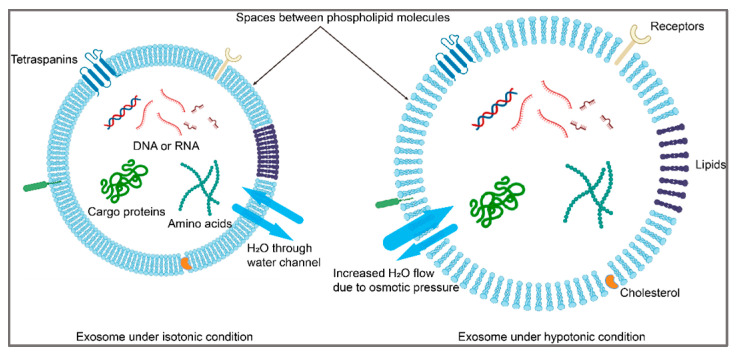
Schematic diagram of exosome structures under isotonic (left) and hypotonic conditions, respectively. The spaces between phospholipid molecules under hypotonic conditions increase as a response to osmotic pressure differences on both sides of the membrane.

For simplicity, exosomes are assumed to maintain a spherical geometry with a uniform distribution of DOX once internalized into them. DOX loading into exosomes occurs through passive diffusion across the exosomal membrane. The loading process in the study was driven by the concentration gradient between the exterior (DOX solution dissolved in PBS) and the interior of the exosome. The osmotic pressure difference across the membrane caused by hypotonic treatment (for example, a quarter-strength osmolarity solution, 1:3 PBS to water) would also have an impact on the loading. Initially, the interior of the exosome is devoid of DOX, while the exterior contains a known concentration of DOX in PBS. To facilitate the derivation of the loading process, several other assumptions were established or acknowledged in this study: (a) internal contents/cargoes of exosomes within proximity to the membrane (~50 nm) do not significantly impact the diffusion process of DOX; (b) the permeability of the exosome membrane to DOX is affected under hypotonic conditions, facilitating an enhanced diffusion of DOX into the exosome (it was simply assumed here that permeability varies linearly with the volume of vesicles); and (c) the osmotic pressure of the DOX solution outside the exosomes remains almost unchanged by the PBS while DOX diffuses in, considering the fact that the concentration of salts in PBS is much higher than that of DOX dissolved. Typically, 1x PBS solution has a final concentration of 150 mM of various components added up, while the concentration of DOX in this study was less than 1 mM. The initial osmotic pressure difference between the interior/exterior of exosomes induces swelling of vesicles, which increases their volumes and membrane permeability, thereby promoting the DOX influx. As DOX accumulates inside the exosomes over time, the osmotic pressure difference between the interior/exterior decreases, subsequently affecting the rate of DOX loading. Figure 1 below exhibits the difference in the membrane structure of exosomes under isotonic and hypotonic conditions, respectively.

The mathematic model of loading DOX into exosomes in this work considered both the concentration gradient of DOX across the exosome membrane and the osmotic pressure difference as driving forces. Additionally, it accounts for the decrease in osmotic pressure difference and volume change of exosomes over time as DOX accumulates inside. A size-dependent permeability factor was also introduced to incorporate the impact of loaded molecular size under hypotonic conditions (smaller molecules would have higher permeability in general). Given the additional osmotic pressure-driven force, the rate of loading DOX could be described by an augmented version of Fick’s law to include the effects of both concentration gradient and osmotic pressure difference. The net flux (*J*) of DOX across the exosome membrane shall be modeled as

(1) $$J=P(V)\cdot (C_{out}-C_{in})$$

(2) $$P(V)=\frac{P_{0}}{1+\alpha d}\cdot (1+\gamma \frac{V_{t}-V_{0}}{V_{0}})$$

where *P(V)* is the membrane permeability of exosomes to molecules; *C_out_* and *C_in_* are the concentration of DOX outside and inside the exosome, respectively; *P*_0_ is the base permeability of the membrane without size consideration; *α* is a constant representing the degree to which the size of molecules influences permeability; *d* is the average size/diameter of loaded molecules; *γ* is a constant that describes the degree to which the permeability increases as the volume of exosomes increases; *V*_0_ is the initial volume of the exosome before hypotonic treat; *V_t_* is the volume of the exosomes, which is a function of time.

The rate of change of DOX concentration inside the exosome can then be described by

(3) $$\frac{dC_{in}}{dt}=\frac{J}{V}$$

Since the volume, *V*, of exosomes increases due to the osmotic pressure difference between the exterior/interior of exosomes, the volumetric change can be approximated by

(4) $$V_{t}=V_{0}\cdot \left(1+\beta \left({∆\Pi }_{0}-{∆\Pi }_{t}\right)\right)$$

where *β* is a constant reflecting the compliance of exosomes to volume changes due to osmotic pressure; Δ*Π*_0_ and Δ*Π_t_* are the osmotic pressure differences between the osmolarity of treating solution (low strength) and interior osmolarity of exosomes at *t* = 0 and at time *t*, respectively; Δ*Π_t_* equals to Δ*Π*_0_ at *t* = 0. Thus, the relationships between the concentration of DOX loaded into exosomes during the process can be rearranged as below by combining and solving Equations (1)–(4):

(5) $$\frac{\mathrm{d}C_{in}}{\mathrm{d}t}=\frac{P_{0}}{V_{0}(1+\alpha d)}\cdot \frac{1+\gamma \beta \left({∆\Pi }_{0}-{∆\Pi }_{t}\right)}{1+\beta \left({∆\Pi }_{0}-{∆\Pi }_{t}\right)}\cdot (C_{out}-C_{in})$$

## 2. Methods and Materials

### 2.1. Cell Cultures

This study involved 2 cell lines, NCI-N87 (CRL-5822, stomach tissue, ATCC, Manassas, VA, USA) and A549 (CCL-185, lung tissue, ATCC), purchased from ATCC. The fetal bovine serum (30-2020, ATCC) used in the culture was treated with ultracentrifuge to remove any residual exosomes during the production process. Both cell lines were thawed in a water bath set at 37 °C for 1 min and then transferred into a centrifuge tube containing 9.0 mL complete cell culture medium and spun at 125× *g* in a centrifuge (Rotor F-35-6-30, 5430, Eppendorf, Enfield, CT, USA) for 5 min to remove the dead cells. The cell pellets were resuspended with the complete culture medium and dispensed into a 75 cm^2^ culture flask (T-75 flask; MSPP-90076, VWR, Radnor, PA, USA). Cells were cultured in a humidified incubator (MCO-19AIC, SANYO, Osaka, Japan) at 37 °C, with supplied 5% CO_2_ and 95% air constantly. Media were then collected and re-supplied every 2~3 days. The complete cell culture medium was prepared by adding RPMI-1640 (30-2001, ATCC) and F-12K (30-2004, ATCC) with 10% of the abovementioned fetal bovine serum (FBS) and 1% of penicillin-streptomycin (30-2300, ATCC) for NCI-N87 and A549 cell lines, respectively.

The cells were stored in liquid nitrogen vapor after harvesting them when the flask reached above 90% confluency. The medium was collected for the study after aspirating it from the cell culture flask. The flasks were rinsed with 10 mL Dulbecco’s phosphate buffered saline (30-2200, ATCC), and 2~3 mL of Trypsin-EDTA solution (30-2101, ATCC) was added to remove any traces of serum that contains trypsin inhibitor for 5 min. Then, 6~8 mL of complete growth medium was used to resuspend the cells. The cell suspension was transferred to a centrifuge tube and spun at 125× *g* for 5 min. The cell pellets were collected and added into a cryovial (5000-1020, Thermo Fisher Scientific, Rochester, NY, USA) containing complete culture medium with a 5% (*v*/*v*) Dimethylsulfoxide (4-X, ATCC) after aspirating out the supernatant medium.

### 2.2. Exosome Isolation and Characterization

Exosomes were isolated from the cell culture medium by a size-exclusion chromatography (SEC) method using the commercially available columns (qEVoriginal/35 nm, Izon, Medford, MA, USA). The culture medium was thawed completely at room temperature before isolation. Cell debris and other large contaminants in the medium were first removed with a syringe filter (Millex-GP Filter, 0.22 µm, MilliporeSigma, Burlington, MA, USA) by introducing the medium through it. The filtered medium of 500 μL was then loaded into an Izon column for exosome separation, following the standard operating procedure according to the instructions from the vendor. During the whole process, phosphate-buffered saline (1x PBS, Thermo Fisher Scientific, Rochester, NY, USA) was used as medium buffer for the column equilibrium, sample loading, and elution, column wash stages. Isolated exosomes were kept in the vials and stored in the freezer at −20 °C for future characterization and experiments.

Nanoparticle Tracking Analysis (NTA) was performed using Nanosight NS300 (Malvern Panalytical, Malvern, Worcestershire, UK) to measure size and concentration of EV nanoparticles. Samples were diluted in sterile DPBS to achieve a concentration within optimal range of detection (10^7^–10^9^ particles/mL). For each sample, five particle tracking videos were captured for 60 s each at camera level 14. Flow was administered at a constant speed of 25 with automated syringe. Data were processed by Nanosight software and adjusted to the initial dilution factor. Concentration was expressed in particles/mL and mean size in nm.

To assess heterogeneity in tetraspanin surface marker distribution in EV samples, single-particle interferometric reflectance imaging sensing (SP-IRIS) was performed with the ExoView R100 system and ExoView Human Tetraspanin Kit (NanoView Biosciences, Boston, MA, USA). ExoView chips coated with anti-CD9, anti-CD81, and anti-CD63 capture antibodies were pre-scanned according to generate baseline measurements of pre-adhered particles before adding sample (ExoView Human Tetraspanin Kit NanoView Biosciences, Boston, MA, USA). EV samples were diluted in provided incubation solution, and diluted sample was loaded onto the pre-scanned chip and incubated overnight at room temperature in a sealed 24-well plate. After incubation, anti-CD9 CF488, anti-CD63 CF647, and anti-CD81 CF555 detection antibodies were added to chip, incubated for 1 h, washed, and imaged using the ExoView R100 reader and ExoView Scanner 3.0 acquisition software according to the manufacturers’ instructions.

Transmission electron microscopy (TEM) was used to characterize the morphology and size of the isolated exosomes following a standard procedure. A 4% paraformaldehyde solution (Sigma Aldrich, Burlington, MA, USA) was used to fix the exosome samples on the grid substrate. Moreover, 100 μL of samples was pipetted on a 300-mesh copper grid (Electron Microscopy Sciences, Hatfield, PA, USA) and incubated for about 5 min for the absorption of exosomes on the supporting film. The excess volume on the grid was removed with a pipette. Then the grid was followed by a wash with distilled water and stained with uranyl acetate solution (Electron Microscopy Sciences, Hatfield, PA, USA). The grid was washed again with distilled water and dried at room temperature. An electron microscope system (TF20 High-resolution EM, FEI, Hillsboro, OR, USA) was operated to image the samples.

### 2.3. SERS Substrate Fabrication

The SERS substrate was fabricated according to our previously reported method [43], based on the polystyrene sphere lithography. Please refer to the Supplementary Materials for more details.

### 2.4. Transferring Graphene

Single-layer graphene was purchased from the vendor in the form of square films (10 mm × 10 mm) on a copper foil with a layer of PMMA coated on (ME0406, MSE Supplies, Tucson, AZ, USA). The transferring process was adapted from the literature with slight modifications [44,45]. Please refer to the Supplementary Materials for the detailed process.

### 2.5. Scanning Electron Microscopy (SEM)

**Figure 2:**
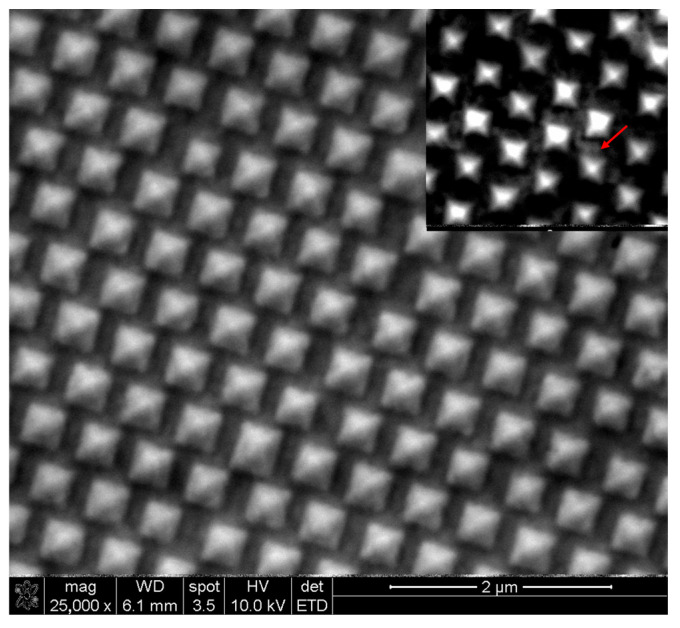
SEM picture of gold nanopyramids substrate. Scale bar: 2 μm. The inset illustration shows the substrate covered with graphene, where the gray shaded areas indicate graphene wrinkles on the surface, marked with red arrow.

The SERS substrate was characterized using SEM imaging with a FEI Nova NanoSEM 230 microscope (FEI, Hillsboro, OR, USA) at an accelerating voltage of 10 kV, with the ETD detector at a working distance of 6.1 mm, shown in Figure 2.

### 2.6. Incubating Drugs with Exosomes

Doxorubicin (DOX) was purchased from the vendor (D-4000, LC Laboratories, Woburn, MA, USA). DOX solution was prepared at different concentrations using 1x PBS as solvent. Notably, 100 μL of isolated exosomes (~3.26 × 10^10^ particles/mL) from NCI-N87 cell line after thawing at room temperature was mixed with 200 μL of DOX solution (0.1 and 0.5 mg/mL, respectively) for the drug loading. The mixture was incubated at 37 °C for 1h with shaking at 125 PRM in an incubator (MaxQ 4000 Benchtop Incubating/Refrigerating Shakers, Thermo Scientific). After the incubation, the admixture was spun by ultracentrifuge (Optima™ TLX Ultracentrifuge, Beckman Coulter, Indianapolis, IN, USA) at ×110,000 g for 90 min to remove the supernatant with unloaded DOX. The supernatant was collected for UV-Vis measurement while the exosome pellets were re-suspended in 1x PBS for the following SERS measurement (~40 loaded exosomes were measured for the study) and other typical exosomal characterizations.

### 2.7. Loading DOX Under Hypotonic Conditions

A quarter-strength (1:3 PBS to water) hypotonic medium with a lower osmolarity of 75 mOsm/L was prepared for the treatment. Isolated exosomes derived from A549 cell line after thawing at room temperature were exposed to the prepared low osmolarity medium for 3 different durations (0.5, 1, and 2 h, respectively) for comparison. Excess 1x PBS solution with DOX dissolved was then added to the samples to equilibrate the osmotic pressure to isotonic condition for 30 min at 37 °C with shaking at 125 PRM. Two different concentrations of DOX (0.1 and 0.5 mg/mL, respectively) were tested in the experiment. The admixture was followed by ultracentrifugation (Optima™ TLX Ultracentrifuge, Beckman) at ×110,000 g for 90 min to remove the supernatant with unloaded DOX. The exosome pellets were re-suspended in 1x PBS for the following SERS measurement (~50 loaded exosomes were measured). Table 1 below presents the various hypotonic conditions applied to purified exosomes during treatment.

### 2.8. Raman Spectroscopy

A droplet of ~5 μL of the sample exosomes was pipetted on the SERS substrate with graphene covered. The droplet was allowed to completely dry in the desiccator within ~15 min, which dries the exosomes to stationary phase, bringing both the membrane and internal components into close proximity with the substrate surface. The substrate with dried sample was carried to a Raman spectrometer (inVia™ confocal Raman microscope, Renishaw, West Dundee, IL, USA) for an immediate SERS data acquisition controlled by WiRE 4.4 software. A 785 nm laser was selected for the biological sample measurement because it has less chance of causing sample overheating with the power of 5 mW, as well as it excites less fluorescence background compared to other shorter wavelength lasers. The map scanning function incorporated by the software enables the scouting and SERS measurement of the single vesicles. To seek the positions of single vesicles, a large square map, typically an area of 300 μm × 300 μm, with a step size of 10 μm was set up for scouting under Raman static mode. After scouting the rough positions of single vesicles, a small area mapping under Raman extended mode, typically mapping size set with a 2 × 2 grid while the step size set at 1 μm, was carried out to get high signal-to-noise ratio spectra. The laser power and exposure time for scouting vesicles were set at 50% and 0.1s under static mode, while for obtaining high signal-to-noise ratio spectra, the parameters were set at 1% and 10s under extended mode, respectively, to reduce the chance of sample overheating. A spectra-selecting lab-developed program was then run to traverse all data after acquisition to pick out the spectra to avoid any noisy spectra, cosmic rays, and potential strong background fluorescence.

## 3. Results

### 3.1. Characterization of Exosomes

**Figure 3:**
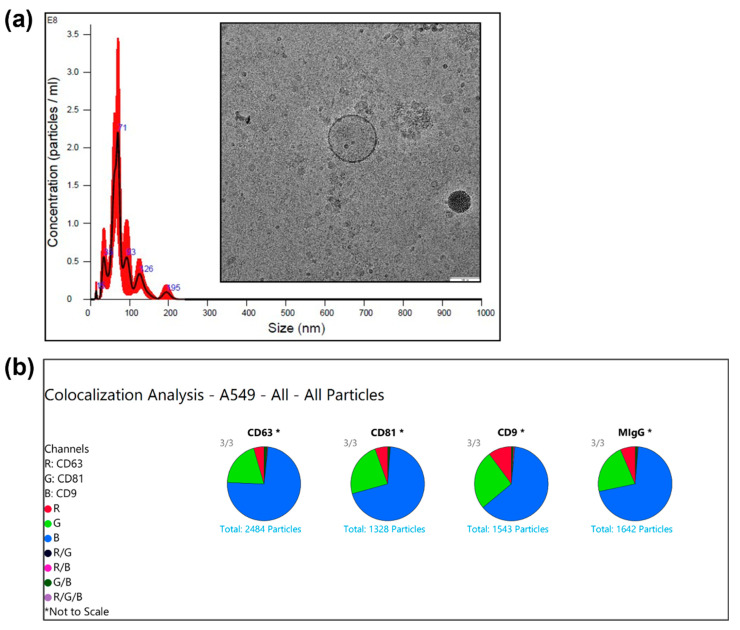
(**a**) Nanosight nanoparticle tracking analysis results of A549 cell line derived exosomes and a typical TEM image of an exosome with double membrane structure revealed clearly. Scale bar: 100 nm. (**b**) Exoview analysis of chosen characteristic surface biomarkers on exosomes from A549 cell line.

After isolation via size exclusion chromatography (Izon), exosomes were characterized using NTA with Nanosight for size distribution and concentration. Figure 3a presents a typical graph of nanoparticle size distribution plot from A549 cell line-derived exosomes along with a TEM image of an exosome with its double-layer membrane structure clearly revealed. The several marked peaks in the graph indicate that there are concentrations of particles at those specific sizes, with a prominent peak at around 71 nm and other smaller peaks at around 35, 93, and 126 nm while Figure 3b displays the colocalization analysis of surface markers of exosomes isolated from A549 cell lines, including CD9, CD63, and CD81 tetraspanins by ExoView Tetraspanin Kit.

### 3.2. SERS Measurements of DOX and Peak Changes Due to Graphene

**Figure 4:**
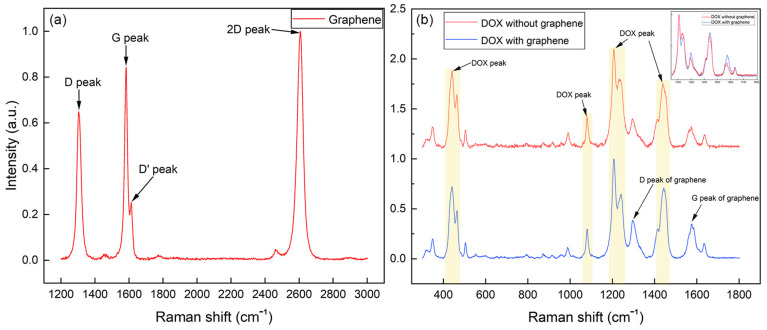
SERS spectra of (**a**) single layer graphene on gold nanopyramid substrate; (**b**) DOX (400 ppm) absorbed on single-layer graphene covered gold nanopyramid substrate and DOX without graphene on the substrate. Inset: Superimposed SERS spectra of both from Raman shift 1150 to 1800 cm^−1^ to highlight the D and G peaks of graphene.

**Figure 5:**
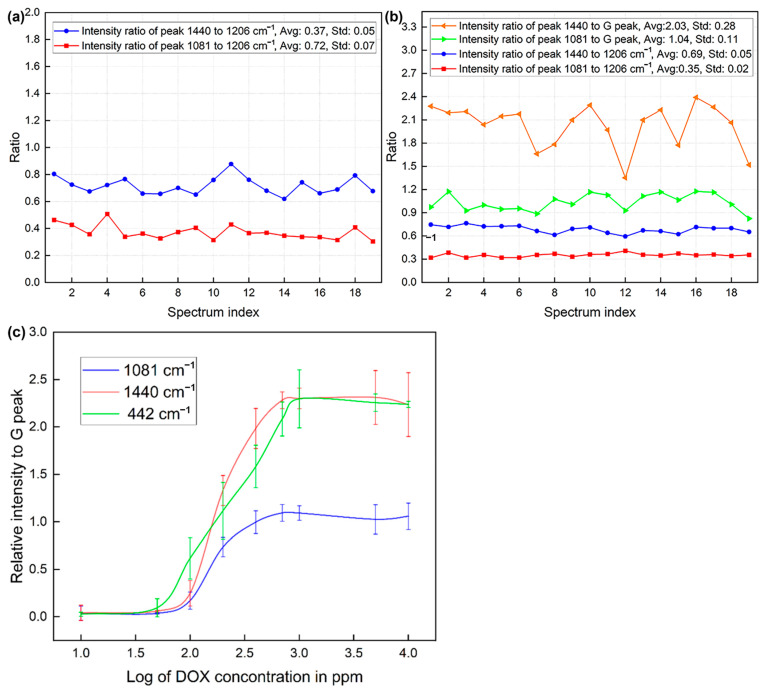
The plots of ratios between peaks of DOX without graphene (**a**) and with graphene (**b**). In plot (**b**), 1081 and 1440 cm^−1^ peaks were also plotted against the G peak of graphene; (**c**) The plot of relative intensity of three Raman peaks of DOX against the G peak of graphene at different concentrations in a logarithm scale.

The exosome drying process before SERS measurement allows exosomes to reduce size and turn into a stationary phase, resulting in their membrane structures and internal entities being positioned in close proximity to the substrate. This dried configuration creates a compact layer that is optimally positioned to leverage the electromagnetic (EM) field enhancement provided by the SERS substrate. The proximity of the exosomes to the substrate ensures significant amplification of the SERS signals. The stationary exosomes enhanced signal stability and reproducibility during SERS measurement. Furthermore, recent studies have shown that the effective enhancement range of SERS can extend up to 20–30 nm, depending on the size, shape, and material composition of the substrate [46,47,48]. This enhancement range is sufficient to encompass the dried exosome samples, enabling efficient interaction with the localized EM fields. The compatibility of the exosome dimensions with the effective SERS range highlights the suitability of this method for detailed analysis. Figure 4a depicts the characteristic SERS spectrum of single-layer graphene enhanced on our nano pyramid gold substrate, with its iconic D, G, D’, and 2D peaks labeled next to their corresponding Raman shift. The typical Raman shifts of these characteristic peaks acquired with our substrate are approximately located at ~1304, 1582, 1613, and 2606 cm^−1^, respectively. Figure 4b demonstrates the SERS spectrum of DOX molecules adsorbed onto our graphene-covered substrate, while the upper spectrum is the SERS spectrum of DOX without graphene. The characteristic peaks of DOX and graphene were marked out in different colors, respectively. In this work, 3 characteristic peaks were chosen for a quantitative study, including 442, 1081, and 1440 cm^−1^ in Raman shift because these peaks have strong Raman intensity and a sufficient shift distance from the D and G peaks of graphene, which would prevent a potential convolution issue when conducting the quantitative study. To investigate whether the presence of graphene affects the changes in the Raman peaks of DOX, this study examined the ratio between different peaks of DOX in the presence and absence of graphene. A solution of DOX in PBS at 400 ppm was dried on the gold nanopyramid substrate without and with graphene separately and subject to SERS measurements. The plots in Figure 5a,b compare the internal ratios of peak intensity of DOX with and without graphene. As indicated, the average peak ratios of Raman shift 1081 cm^−1^ over 1206 cm^−1^ and 1440 cm^−1^ over 1206 cm^−1^ in the absence of graphene were 0.37 (±0.05) and 0.72 (±0.07), respectively. While the graphene was applied on the substrate, the two ratios were 0.35 (±0.02) and 0.69 (±0.05), respectively. In addition, the ratios of 1081 and 1440 cm^−1^ peaks against the G peak were 1.04 (±0.11) and 2.03 (±0.28), respectively. It is obvious that the presence of graphene had a negligible impact on the selected Raman peaks of DOX, which enhanced the credibility of single-layer graphene as the internal gauge of the quantification ability of DOX. The ratios of the DOX peak against the G peak, however, had a larger fluctuation compared to the internal DOX peak ratios, which is led by the fact that DOX also presents a weak peak around the G peak position. Still, it is not hard to conclude that the presence of graphene generates little interference with DOX Raman peaks. Figure 5c depicts a graph plotting the relative intensity of these 3 Raman peaks of DOX (concentration in parts per million (ppm)) against the G peak of graphene in a logarithm scale. At lower DOX concentrations (Log [DOX] ≈ 1.5–2.5), the relative intensities of the Raman peaks are low for all three peaks. There is a sharp increase in the relative intensity of the 1440 cm^−1^ and 442 cm^−1^ peaks when the concentration of log [DOX] in the range between 2.5~3.5, indicating that these Raman modes become more prominent as the concentration of DOX increases than the peak of 1081 cm^−1^. The graph indicates that the 1440 cm^−1^ and 442 cm^−1^ Raman peaks of DOX show a strong, concentration-dependent increase in intensity relative to the G peak of graphene. This suggests that these two peaks could be particularly sensitive to changes in DOX concentration while the 1081 cm^−1^ peak, also increasing with concentration, shows a more gradual and less pronounced response to the concentration changes. This plot can be used to determine the concentration of DOX based on the intensity of these Raman peaks relative to the graphene G peak, especially within the concentration range where the intensity response is most linear.

### 3.3. SERS Measurements of DOX Loaded into Exosomes

**Figure 6:**
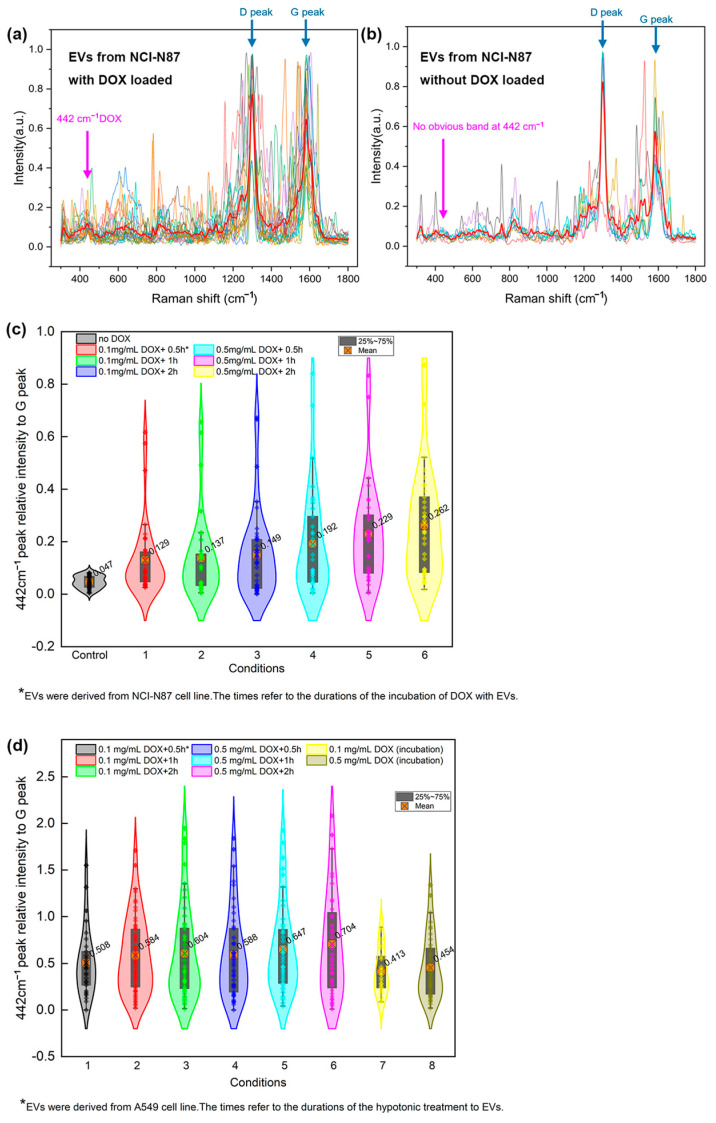
(**a**,**b**) Superimposed SERS spectra of exosomes derived from NCI-N87 cell line with DOX loaded (**a**) and without (**b**). The red bold curves are the averaged plots for both situations and the different colored thin curves in the spectra represent individual measurements collected from various exosome. (**c**) The violin distribution diagram of DOX loaded into exosomes derived from NCI-N87 cell line under different incubation conditions in terms of the 442 cm^−1^ peak intensity over the G peak of graphene, and (**d**) the violin distribution diagram of DOX loaded into exosomes derived from A549 cell line under different hypotonic treating conditions.

Figure 6a,b plot multiple SERS spectra of exosomes loaded with and without DOX, respectively, using Raman shift at 442 cm^−1^ as the indicator. The red bold curves are averaged plots from all acquired SERS spectra. A violin diagram of the relative intensity of loaded DOX peak at 442 cm^−1^ to graphene G peak in the exosomes derived from the NCI-N87 cell line under different incubation conditions was plotted in Figure 6c using the data extracted from their SERS spectra. Similarly, another violin diagram of the relative ratio of DOX peak at 442 cm^−1^ to G peak in the exosomes derived from the A549 cell line under varied hypotonic treatments was presented in Figure 6d. The mean value was labeled in the figure next to each violin shape and the gray rectangle covered data range from the 25th percentile to the 75th percentile. The DOX loading results in exosomes isolated from NCI-N87 cell line culture by UV-Vis absorption changes, and a fitting curve of the absorption against DOX concentration are provided in the Supplementary Materials for validating the loading process.

## 4. Discussion

The NTA results provided size and concentration information, indicating that the isolated particles are within the exosome size range. Combined with TEM images showing characteristic exosomal morphology, these findings suggest the presence of exosome-like vesicles in the samples isolated from cell lines. The ExoView analysis suggests that within the particles analyzed, there are populations of particles positive for those tetraspanins biomarkers, with a significant count of particles positive for each marker individually, and a smaller subset of particles positive for multiple markers, indicating a degree of colocalization between these molecules. This can imply that the entities carry a mixture of these proteins on their surface, thus, exosomes were verified with the characteristic biomarkers. We acknowledge that exosomes exhibit inherent heterogeneity in size, composition, and cargo capacity, which may influence their drug-loading efficiency. However, the primary goal of this study was to deliver an adequate dose of drugs through exosomes to cells, rather than focusing on the variance of exosomes in the loading process. Our concentration gradient-based drug-loading approach relies on the principle of diffusion, where the drug concentration equilibrates between the external and internal environments of the exosomes. Under ideal conditions, this ensures that the amount of drug loaded into each exosome is primarily determined by the starting drug concentration. That said, we recognize that high-resolution size fractionation techniques would provide valuable insights into size-dependent drug-loading differences. However, traditional methods such as ultracentrifugation and even SEC often lack the precision or yield required for detailed size-dependent analysis. While the current study did not specifically analyze this, our previous research suggests that exosome size subfractions have distinct biochemical profiles [42], potentially impacting their drug-loading potential. To address this, we are conducting a follow-up study utilizing high-resolution fractionation and sensitive analytical techniques to explore the relevance of exosome size subfractions and their biochemical content. This ongoing work aims to optimize exosome-based drug delivery systems by accounting for size-dependent variability.

A comprehensive drug loading and delivery study should encompass a wide range of combinations, including EV loading levels, the duration of EVs exposed to hypotonic conditions, the cell of origin of the EVs, targeted cell types, and more. This is beyond the scope of the current study and will be addressed in future research. Monitoring the expression levels of tetraspanins, instead of conducting cell uptake experiments, serves as an initial step toward evaluating the biological functions of the drug-loaded EVs, considering the following two points: first, tetraspanins are widely recognized and commonly used markers for exosome characterization [49,50]; second, further testing should incorporate the aforementioned factors in the subsequent cell uptake experiments.

It is quite noticeable that Raman shifts of some characteristic peaks of single-layer graphene from our substrate were shifted to a certain degree. For example, the D and 2D peaks of graphene normally appear at 1350 and 2700 cm^−1^ in a typical Raman spectrum [51] while located at ~1304 and 2606 cm^−1^ in this work. However, these peaks may shift to varying degrees under the influence of certain factors, such as the laser excitation wavelength, strain, doping, and the environment in which the graphene is placed [52,53]. For example, the Raman shift of the D peak indicates a strong positive correlation with the laser intensity. According to the literature, the position of the D peak shifts further to the left with a weaker laser power. In our study, the shift in the peaks was mainly caused by the strain induced by the extremely rough surface of the gold nanopyramid substrate and the laser power applied. Furthermore, the shape and symmetry of the 2D peak are direct and powerful evidence of a single-layer graphene structure on our substrate [52,54]. For single-layer graphene, the 2D peak is generally symmetric and extremely sharp, often appearing as a single, Lorentzian-shaped peak while it would lead to a more complex or split peak, reflecting the interaction between layers in a multilayer graphene structure. For instance, the 2D peak of a 2-layer graphene would split into 4 peaks from the original sharp peak due to the phonon dispersion by the coupling between electron and phonon [54]. It is highly recognized that the shape of the 2D peak of graphene is decidedly sensitive to the stacking order and the number of graphene layers. This is because the characteristics of the second-order process with opposite momentum change significantly with the number of graphene layers, which is associated with a second-order process involving two phonon lattice vibrations. The peak at ~1304 cm^−1^ of graphene is known as the D band which is associated with the breathing modes of sp^2^ atoms in rings and is indicative of defects in the graphene lattice [55]. This peak is typically absent or very weak in high-quality, defect-free graphene. However, due to the extreme roughness of the substrate indicated by the SEM photo, the covered single-layer graphene on top would inevitably create numerous folds and wrinkles on the surface [56]. Thus, a relatively strong D band would occur in the SERS spectra without being a surprise. The G band at approximately 1582 cm^−1^ is attributed to the in-plane vibration of sp^2^ bonded carbon atoms which is universal in all graphitic materials [55]. For single-layer graphene, the 2D peak is particularly sharp and has a higher intensity than the G peak with a single Lorentzian shape. This is because there is no interlayer coupling in single-layer graphene, which would otherwise affect the vibrational modes. Therefore, our single-layer graphene on the substrate can be confirmed by the sharp 2D peak presented in the spectrum. In short, the appearance of all characteristic peaks of graphene and the sharp, symmetric shape of the 2D band indicates that we have successfully transferred high-quality single-layer graphene onto the substrate. In Figure 4b, the presence of peaks attributed to graphene in the spectrum with DOX indicates the coexistence of the graphene and DOX on the substrate, further illustrating that the graphene has some level of defects with the D peak and confirms the in-plane vibration characteristic of graphene with the G peak.

We have verified that the single-layer graphene on the substrate allows us to conduct quantification of SERS with DOX molecules according to Figure 5. The Raman shifts including 442, 1081, and 1440 cm^−1^ were plotted to reveal the changes in their intensity relative to the G peak of graphene with increasing DOX concentration. The G peak was used as an internal gauge because it is a prominent and stable feature in the Raman spectrum of graphene. Each line shows an S-shaped curve, which is typical for a binding isotherm. This suggests that there is a saturation point beyond which increasing the DOX concentration does not lead to a significant increase in the Raman signal. This could be indicative of a maximum adsorption capacity of DOX on the graphene surface. The plateau in each curve suggests that a saturation point is reached, beyond which the amount of DOX adsorbed onto the graphene does not significantly change with increased concentration. This is where all available binding sites on the graphene surface are occupied. The different slopes of the curves prior to reaching saturation may suggest the sensitivity of each vibrational mode to the DOX concentration. For instance, the 1440 cm^−1^ line rises more steeply than the other two lines, which could mean that the 1440 cm^−1^ Raman shift is more sensitive to changes in DOX concentration at lower levels. The graph could be used to quantify the DOX molecules in proximity to the graphene layer since it provides a visual and quantitative method to determine the concentration of absorbed DOX on the surface which would serve as the basis of quantification of loaded DOX into individual exosomes.

Among three selected DOX peaks for quantification study, the 442 cm^−1^ peak was ultimately selected as the standard for quantification because the other two peaks (1081, and 1440 cm^−1^) may overlap with peaks produced by tissues or cellular structures at the same positions, specifically ν_1_CO_3_^2−^, ν_3_PO_4_^3−^, ν(C-C) skeletal of acyl backbone in lipid generating a peak at 1081 cm^−1^ while CH, CH2, and CH3 deformation vibrations at 1440 cm^−1^, according to the literature [57]. However, the closest potential peak to the 442 cm^−1^ peak is the N-C-S bonding stretch by thiocyanate at 445 cm^−1^, which has a 3 Raman shift distance to the target peak. In this study, we utilized exosomes from two different cell lines (NCI-N87 and A549) to demonstrate the general applicability of our osmotic pressure-based drug-loading method across diverse exosome sources. The observed differences in DOX loading efficiency between the two exosome types highlight the inherent variability in drug-loading capacity, which is influenced by the unique biological specificities of each cell line. Importantly, the lack of an order-of-magnitude difference in loading efficiency further underscores the broad applicability of our method to various exosome sources. While these differences are intriguing, the biochemical mechanisms underlying them remain to be fully elucidated. A detailed exploration of these factors, however, is beyond the scope of the current study and will be the focus of future investigations. Several findings can be drawn by summarizing the drug loading results from the plots: (i) EVs derived from different cell lines exhibited varying degrees of loading capacity, as reflected in the average relative intensity of the 442 cm^−1^ peak from the EVs of the two cell lines. Specifically, for NCI-N87-derived EVs, the average relative intensity of the 442 cm^−1^ peak is 0.149 and 0.262 at initial DOX concentrations of 0.1 and 0.5 mg/mL, respectively, while for A549-derived EVs, the values are 0.413 and 0.454 under the same conditions; (ii) The effect of different incubation times (0.5, 1, and 2 h) of EVs, when DOX was at the same concentration, on the loading outcome is not significant, showing only slight improvement. However, longer incubation times tend to slightly increase the variability in loading results; (iii) Hypotonic treatment significantly enhances loading efficiency. At 0.1 mg/mL DOX, the average 442 cm^−1^ peak intensity increased by 0.095 (23.0%), 0.171 (41.4%), and 0.191 (46.2%) for treatment times of 0.5, 1, and 2 h, respectively. At 0.5 mg/mL DOX, the average 442 cm^−1^ peak intensity increased by 0.134 (29.5%), 0.193 (42.5%), and 0.250 (55.1%) at the same treatment times; (iv) The loading results are not particularly sensitive to drug concentrations and treatment times when EVs underwent hypotonic treatment, exhibiting only slight improvements; and (v) In every single case, some EVs possessed particularly high peak intensities under their respective conditions, potentially representing a subgroup of EVs that are particularly conducive to drug loading. These EVs warrant further investigation, as they could be key factors in enhancing loading efficiency. Future research should focus on the precise identification and isolation of these EVs for more detailed analysis. The above results indicate that hypotonic treatment can effectively enhance the loading efficiency of EVs, allowing for more effective and faster drug loading compared to standard incubation methods. The lack of sensitivity of the loading effect to drug concentration and incubation time suggests that 0.1 mg/mL DOX may already be a relatively high loading concentration, and most of the drugs were likely loaded within the first 0.5 h.

Compared to conventional characterization methods of loaded EVs, like UV-Vis, which do not provide information at the single exosome level, potentially leading to missed critical insights for further investigation, our SERS-based technique enables direct detection and quantification of DOX loading at the single exosome level, providing an additional dimension of information. As shown in Figure 6c, at lower DOX concentrations, varying incubation times have a limited impact on overall loading efficiency. However, longer incubation times allow the majority of exosomes to reach loading equilibrium, reducing variability in single exosome loading levels. Shorter incubation times may result in exosome overloading, which explains the larger fluctuations in individual loading levels at 0.5 h. At higher DOX concentrations, achieving exosome loading equilibrium requires more time, as evidenced by the continued fluctuations observed after two incubation periods (0.5 h and 1 h). These findings set the stage for further exploration of the optimal DOX loading concentration and incubation time for exosomes.

## 5. Conclusions

In this work, we successfully developed a SERS-based monitoring platform for enhanced drug-loading capacity of exosomes, using doxorubicin as a model molecule. This system enables the precise detection and quantification of drugs in individual vesicles, utilizing single-layer graphene as an internal gauge of plasmon resonance intensity allowing for quantitative measurements of DOX using SERS. Our findings demonstrate that applying a hypotonic condition significantly increases drug loading efficiency without compromising the integrity of the exosomes. These results pave the way for advancing exosomes as practical drug carriers in medical applications. In conclusion, our study demonstrates the potential of SERS for analyzing DOX within exosomes, facilitated by the drying-induced proximity of exosomal components to the SERS-active substrate. However, we acknowledge the complexities in pinpointing the exact localization of DOX within exosomes and recognize that further characterization using complementary techniques could provide additional insights. Future research should focus on employing advanced imaging and analytical methods to precisely determine the intravesicular distribution of DOX, thereby enhancing the accuracy and reliability of SERS-based analyses in this context. Moving forward, we believe that the ultimate goal in the journey of establishing this new technique of drug loading and measurement is to conduct extensive cell culturing experiments to examine the uptake of selected EVs by targeted cancer cells. This ongoing work will address the complexities of these biological processes and further validate the potential of exosomes in drug delivery.

## Figures and Tables

**Table 1:** Sample treatment under hypotonic conditions.

Samples	Osmolarity	Treating Time (h)	DOX Conc. (mg/mL)
**1**	A quarter-strength (1:3 PBS to water)	0.5	0.1
**2**		1	0.1
**3**		2	0.1
**4**		0.5	0.5
**5**		1	0.5
**6**		2	0.5
**7**	Isotonic (1x PBS)	n.a.	0.1
**8**		n.a.	0.5

## Data Availability

The original contributions presented in this study are included in the article/Supplementary Material. Further inquiries can be directed to the corresponding author.

## Supplementary Materials

The following supporting information can be downloaded at: https://www.mdpi.com/article/10.3390/bios15030141/s1, Figure S1: UV-Vis absorption fitting curve of DOX; Figure S2: FDTD simulated x-z and x-y views of the electric field amplitude distribution for incident light; Figure S3: SERS intensity maps generated with respect to nucleic acid, lipid, and protein from the same data spot; Figure S4: SERS measurement of DOX loaded into exosomes. 3 replicates of exosomes under the same condition (incubated with 0.1 mg/mL DOX for 1 h) were performed according to the protocol and subject to SERS measurement. Table S1: Table of UV-Vis readings of loading of DOX into exosomes derived from NCI-N87 cell line; Table S2: Mean value of relative DOX peak intensity and corresponding standard deviation from the 3 groups of exosomes measured by SERS; Equation S1: Theoretic calculation of average spacing of exosomes.
