# Supplementary material for: On the Feasibility of SERS-Based Monitoring of Drug Loading Efficiency in Exosomes for Targeted Delivery
Source: Biosensors (Basel). 2025 Feb 23;15(3):141. doi: 10.3390/bios15030141 (PMC11939968; doi:10.3390/bios15030141)
Supplement: Supplementary file 1 [file biosensors-15-00141-s001.zip › biosensors-3463215-supplementary.pdf]

Supplemental materials of "On the Feasibility of SERS-Based Monitoring of Drug Loading Efficiency in Exosomes for Targeted Delivery"

Jun Liu, Siddharth Srivastava, Tieyi Li, Faycal Moujane, John Lee, Yiqing Chen, Huinan Liu, Sophie Deng, Ya-Hong Xie

1. SERS substrate fabrication

Polystyrene spheres with a diameter of 500 nm (Alfa Aesar) were first compressed to form a monolayer on the surface of water in a Langmuir–Blodgett trough. This compression induced these spheres to closely pack into hexagonal patterns. Four-inch (100) oriented Si wafers (MSE Supplies) were treated with thermal oxidation to generate a SiO_2 layer about 50 nm in thickness on the wafer surface. Then the polystyrene sphere patterns were transferred onto those SiO_2/Si wafers by slowly pulling the wafers out of the sphere-covered trough. The wafers with spheres covered were dry etched by O_2 plasma etching (Oxford Plasmalab 80 Plus) for 50 s at 200 W power to reduce the size of the spheres to about 250 nm in diameter. A chromium (Cr) film of 50 nm in thickness was deposited on the as-prepared wafers using electron beam deposition (CHA Mark 40). After the deposition of the Cr film, the polystyrene spheres were lifted off by ultrasonically cleaning the wafers in acetone, and the wafers were thoroughly rinsed with DI water three times. The Cr layer, with small holes caused by lifting the spheres, served as the mask in the subsequent step of etching the exposed SiO_2 layer. The SiO_2 film beneath the Cr film was dry etched by a mixture of plasma of Ar flow (25 sccm) and CHF_3 flow (25 sccm) at 200 W for 2 min (Oxford Plasmalab 80 Plus). The Cr film was then removed by immersing the wafers in a Cr etchant solution. The wafers were followed by etching in a KOH aqueous solution (60 wt%, Sigma Aldrich) for 2 min at 60°C to pattern the inverted pyramidal structures on the wafers as the mold. An HF solution (30%, Sigma Aldrich) was used to etch away the SiO_2 layer before a 200 nm thick gold (Au) film was deposited on the Si mold with pyramidal structure by electron beam deposition (CHA Mark 40). Finally, fast-cured epoxy was applied to peel off the gold layer with pyramidal structures from the mold to a supporting wafer substrate as the SERS substrate. The fabricated Au pyramids on the substrate with a base length and height both ~200 nm were utilized to enhance the Raman signal of absorbed molecules/exosomes on the surface.

2. Transferring graphene

A $FeCl_3$ solution with a concentration of 1M was prepared for the etching of the copper substrate. The graphene film on the copper substrate was carefully placed on the $FeCl_3$ solution with the copper substrate facing downward. After the copper was completely

dissolved, the film was transferred cautiously to the surface of a DI water bath to rinse away the residuals. The DI water bath was repeated three times. Subsequently, our SERS substrate was placed beneath the graphene film floating on the water, and the graphene was carefully scooped onto the substrate from below. Then the substrate with graphene was dried at room temperature for 1 h and transferred into a desiccator for complete drying, followed by heating in a vacuum environment at 180°C for 1 h. The substrate was soaked in an acetone bath at 50°C for 1 h to remove the PMMA layer, followed by rinsing with isopropyl alcohol three times. Finally, the substrate was dried again in a desiccator before further use.

3. UV-Vis absorption results of DOX loaded into exosomes

Table 1 presents the DOX loading results into exosomes isolated from the NCI-N87 cell line culture under different circumstances determined by UV-Vis absorption changes. UV-Vis was initially employed as a preliminary check for drug loading both qualitatively and quantitatively; however, the technique itself lacks the precision needed for accurate quantification. The absorption results from the UV-Vis spectrophotometer presented in the table seem to be unreliable in quantity, which may be attributed to the following factors and limitations. Specifically, it does not directly measure the DOX loaded but instead infers it from the concentration change of DOX in the solution before and after incubation. Therefore, an additional ultracentrifugation step was performed after loading to remove any unincorporated DOX in the solution. It is speculated that during this step, some EVs may rupture or experience cargo leakage, which would lead to inaccurate measurements of DOX. These cargoes may absorb light at similar wavelengths as the drug, leading to overlapping signals and making it difficult to differentiate. Another less prominent factor is the presence of PBS. Although PBS absorbs very weakly at the UV-Vis wavelength of 480 nm, the PBS salt in the exosome solution would be collected with the unincorporated DOX after ultracentrifugation, potentially contributing to measurement error.

Table S1. Table of UV-Vis readings of loading of DOX into exosomes derived from NCI-N87 cell line

Sample	[DOX] mg/mL	Incubation time (h)	UV-Vis Absorbance (Dilution considered)		Absolute absorption change	Loading percentage (%)
			Before incubation	After incubation		
1	0.05	0.5	0.786 (±0.007)	0.761 (±0.011)	0.025	3.21
2		1		0.746 (±0.015)	0.040	5.13
3		2		0.760 (±0.021)	0.026	3.31
4	0.10	0.5	1.561 (±0.012)	1.506 (±0.022)	0.055	3.62
5		1		1.504 (±0.017)	0.057	3.55
6		2		1.503 (±0.013)	0.058	3.71

4. Fitting curve of UV-Vis absorption of DOX solution

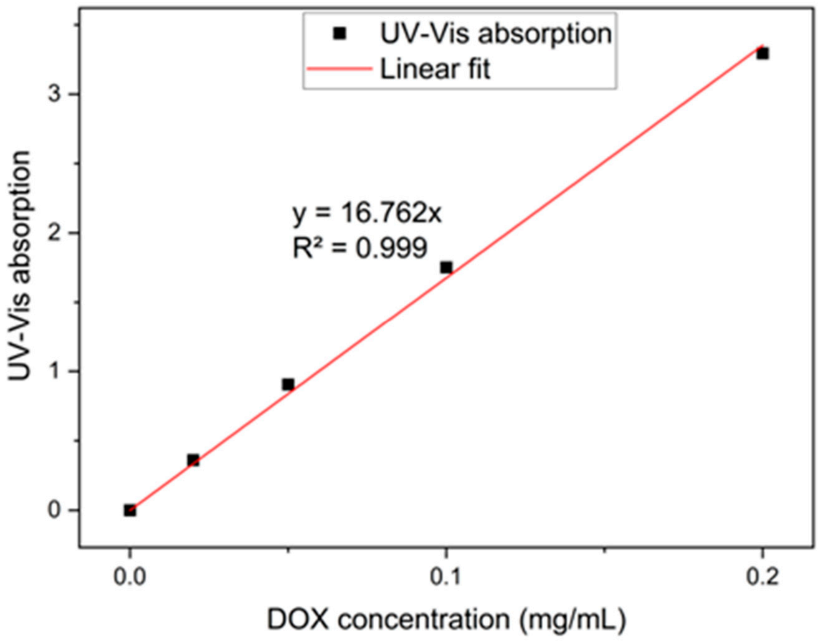

Figure S1. UV-Vis absorption fitting curve of DOX

5. FDTD simulation results of Electromagnetic field of gold nanopyramids substrate

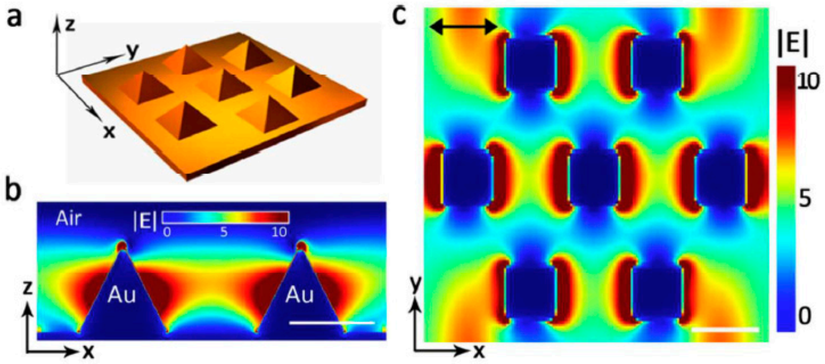

Figure S2. FDTD simulated x-z and x-y views of the electric field amplitude distribution for incident light.[1, 2]

6. Theoretic calculation of exosome area density on the surface

The calculation result is based on the assumptions that the initial concentration of exosome solution is at 5 × 10^8 particles/mL, and a 5 μL droplet that dries into a circular spot with a diameter of 5 mm.

Equation S1. Theoretic calculation of average spacing of exosomes.

$$Total\ exosomes\ in\ sample = 5 \times 10^8 \frac{particles}{mL} \times 5\mu L = 2.5 \times 10^6\ particles$$

$$exosome\ density = \frac{Total\ particles}{area} = \frac{2.5 \times 10^6\ particles}{(2.5\ mm)^2 \pi} = 0.127 \frac{particles}{\mu m^2}$$

$$Average\ spacing\ of\ exosomes = \sqrt{\frac{1}{exosome\ density}} = \sqrt{\frac{1}{0.127 \frac{particles}{\mu m^2}}} = 2.8\ \mu m$$

7. SERS heat mapping of single exosome

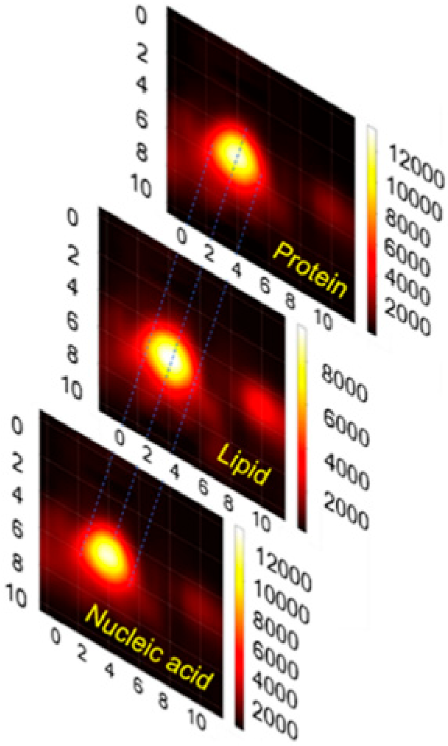

Figure S3. SERS intensity maps generated with respect to nucleic acid, lipid, and protein from the same data spot.[3]

8. Reproducibility test of SERS measurement of DOX loading exosomes

A total of three replicate exosome samples were incubated with a DOX solution (0.1 mg/mL) for 1h (the same condition as the group 2 sample in Figure 6c) and processed according to the procedure described in the manuscript. Approximately 30 exosomes from each replicate were detected and measured using SERS. It demonstrates reasonable reproducibility when comparing the data in tables. The relatively large standard deviation observed in both tables is likely due to the inherent heterogeneity of exosomes. This variability arises because the measurements are based on single exosomes, where each exosome may differ in properties such as size, molecular content, and surface characteristics. Such differences are natural and expected when analyzing individual exosome behavior.

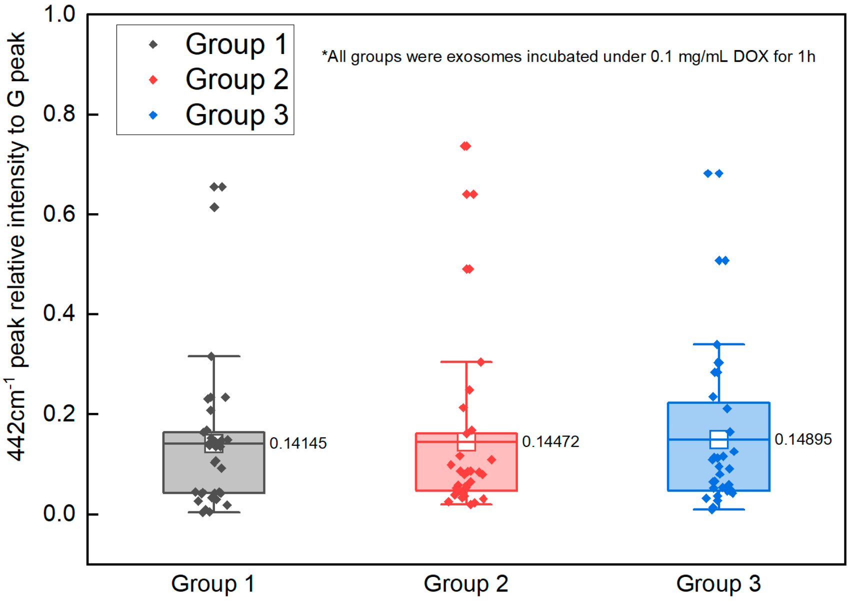

Figure S4. SERS measurement of DOX loaded into exosomes. Three replicates of exosomes under the same condition (incubated with 0.1 mg/mL DOX for 1h) were performed according to the protocol and were subject to SERS measurement.

Table S2. Mean value of relative DOX peak intensity and corresponding standard deviation from the 3 groups of exosomes measured by SERS.

Replicate	Mean relative peak intensity	S.D.
1	0.1415	0.1500
2	0.1447	0.1787
3	0.1490	0.1526

References

1. Wang, P.; Liang, O.; Zhang, W.; Schroeder, T.; Xie, Y. H., Ultra-Sensitive Graphene-Plasmonic Hybrid Platform for Label-Free Detection. *Adv Mater* **2013,** *25* (35), 4918-4924.
2. Wang, P.; Xia, M.; Liang, O.; Sun, K.; Cipriano, A. F.; Schroeder, T.; Liu, H. N.; Xie, Y. H., Label-Free SERS Selective Detection of Dopamine and Serotonin Using Graphene-Au Nanopyramid Heterostructure. *Anal Chem* **2015,** *87* (20), 10255-10261.
3. Liu, Z. R.; Li, T. Y.; Wang, Z. Y.; Liu, J.; Huang, S.; Min, B. H.; An, J. Y.; Kim, K. M.; Kim, S.; Chen, Y. Q.; Liu, H. A.; Kim, Y.; Wong, D. T. W.; Huang, T. J.; Xie, Y. H., Gold Nanopyramid Arrays for Non-Invasive Surface-Enhanced Raman Spectroscopy-Based Gastric Cancer Detection via sEVs. *Acs Appl Nano Mater* **2022,** *5* (9), 12506-12517.
